# Integration of *in vivo* electrophysiology and optogenetics in rodents with PEDOT:PSS neural electrode array

**DOI:** 10.1016/j.xpro.2024.102909

**Published:** 2024-02-29

**Authors:** Young Uk Cho, Ju Young Lee, Ki Jun Yu

**Affiliations:** 1 School of Electrical and Electronic Engineering, Yonsei University, 50 Yonsei-ro, Seodaemungu, Seoul 03722, Republic of Korea; 2 School of Electrical and Electronic Engineering, YU-KIST Institute, Yonsei University, 50 Yonsei-ro, Seodaemungu, Seoul 03722, Republic of Korea

**Keywords:** Neuroscience, Biotechnology and bioengineering

## Abstract

Here, we present a protocol for the fabrication of transparent implantable electrode arrays for integrating optogenetics and electrophysiology. We describe steps for fabricating microelectrodes using the conductive polymer poly(3,4-ethylenedioxythiophene):poly(styrenesulfonate). We then detail procedures for analyzing performance of the electrodes and recording light-evoked neural activities from the transgenic mouse. This protocol utilizes photolithography rather than conventional electrodeposition.

For complete details on the use and execution of this protocol, please refer to Cho et al. (2022).^1^

## Highlights

• Protocol for fabrication of transparent PEDOT:PSS neural electrode arrays
• Introduction of device characteristics interrogation
• *In vivo* electrophysiology with transparent PEDOT:PSS neural electrode array
• Presentation of simultaneous electrophysiology and optogenetics

## Abstract (editor highlights)

Publisher’s note: Undertaking any experimental protocol requires adherence to local institutional guidelines for laboratory safety and ethics.

## Graphical abstract

**Figure:**
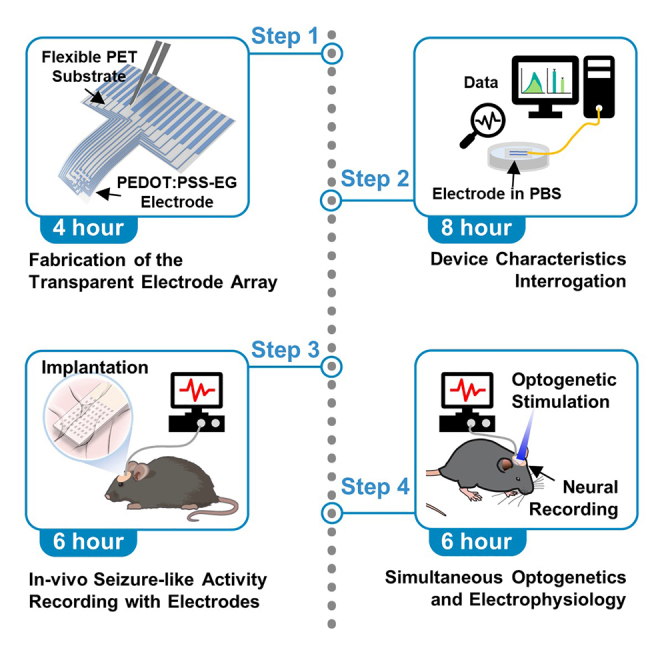

## Before you begin

The method of evaluating complex brain neural activity through electrophysiological recording has become a cornerstone in identifying human behavioral mechanisms for neuroscientists and brain engineers.^2^^,^^3^^,^^4^ Moreover, to alleviate the burden on patients suffering from various neurological disorders (Parkinson’s disease, Alzheimer’s disease, or depression, etc.), electrophysiology plays a significant role in analyzing electrical signals from the brain in finding appropriate therapy.^5^ Independently of electrophysiological approaches, optogenetics, which is controlling neural activity with light, has also received attention as a neuron-specific modulation method.^6^^,^^7^ Genetically modifying specific target neurons, optogenetics realizes changing neural activity with enhanced selectivity than electrical stimulation.^8^^,^^9^

Therefore, the integration of electrophysiology and optogenetics brings significant synergetic effect in real-time monitoring of electrical signals from the brain and neuron-specific modulation.^1^^,^^10^ Simultaneously recording with electrophysiology whether individual neurons and axonal projections modulated by light at a predetermined frequency reduces the difficulty of circuit analysis of complex neurological disorders, which was previously impossible.^11^^,^^12^

However, there are serious problems that arise when electrophysiology and optogenetics are integrated-1. Photoelectric artifact, 2. Limited light transmission.^10^^,^^13^ Photoelectric artifact caused by a neural electrode refers to a small voltage signal by excited free charges when light above a certain energy level is applied to the surface of the metal electrode.^14^^,^^15^ When this voltage signal combines with the small electrophysiological potential from the neural activity, results in contamination of the neural signals.^14^^,^^16^ Additionally, opaque metal electrodes absorb most of the light for neuromodulation, preventing sufficient energy needed for optogenetics from reaching the brain tissue. This limited transmission of light is detrimental to the convergence of electrophysiology and optogenetics in that neural activity would not be activated with the light stimulation threshold.

Transparent, implantable electrode arrays do not absorb but transmit most of the light for stimulation, so there is negligible problem in performing electrophysiology and optogenetic stimulation simultaneously.^17^^,^^18^ Typically, conductive oxides such as indium tin oxide have high optical transparency and electrical conductivity.^19^ However, due to the mechanical brittleness, there is a limitation that it is difficult to be applied to flexible bio-implantable devices. Electrode arrays exploiting 2-dimensional materials such as graphene or carbon nanotubes have served as an important bridge in the fusion of electrophysiology and optogenetics with their optically transparent yet flexible form.^20^^,^^21^^,^^22^ However, the difficulty in fabricating electrodes due to the transfer process of the active electrode layer on the functionalized flexible substrate has remained a major challenge for the application of the carbon-based material.^23^^,^^24^ PEDOT:PSS, a conductive polymer, has high transmittance and superior mechanical properties, and can be expected to have high electrical conductivity due to modification of the molecular structure.^25^^,^^26^^,^^27^^,^^28^

Here, we introduce a protocol for the fabrication of implantable transparent electrodes using PEDOT:PSS. Unlike the existing method of depositing PEDOT:PSS on conventional metal with electroplating, this protocol introduces patterning spin coated PEDOT:PSS using an ultrasonication lift-off. The transparent electrode array consisting single layer of PEDOT:PSS without an additional metal is free from two problems when integrating electrophysiology and optogenetics - photoelectric artifact and limited light transmission. Our protocol presents in detail how to control the neural signals of a genetically modified mouse using a blue laser while performing high-fidelity neural recording with negligible light-induced artifacts. By introducing transparent electrode manufacturing methods and artifact-free electrophysiology with optogenetics, this article presents the potential of conductive polymer-based neural implants as an analyzing tool for neural circuits that had not previously been identified.

### Institutional permissions (if applicable)

The Korea Institute of Science and Technology (KIST) in Seoul, Korea, granted permission for all animal-related procedures, which were carried out in accordance with the ethical guidelines outlined in KIST’s Animal Care and Use Guidelines.

### Preparation of substrate

**Timing: 4 h**

**Figure 1:**
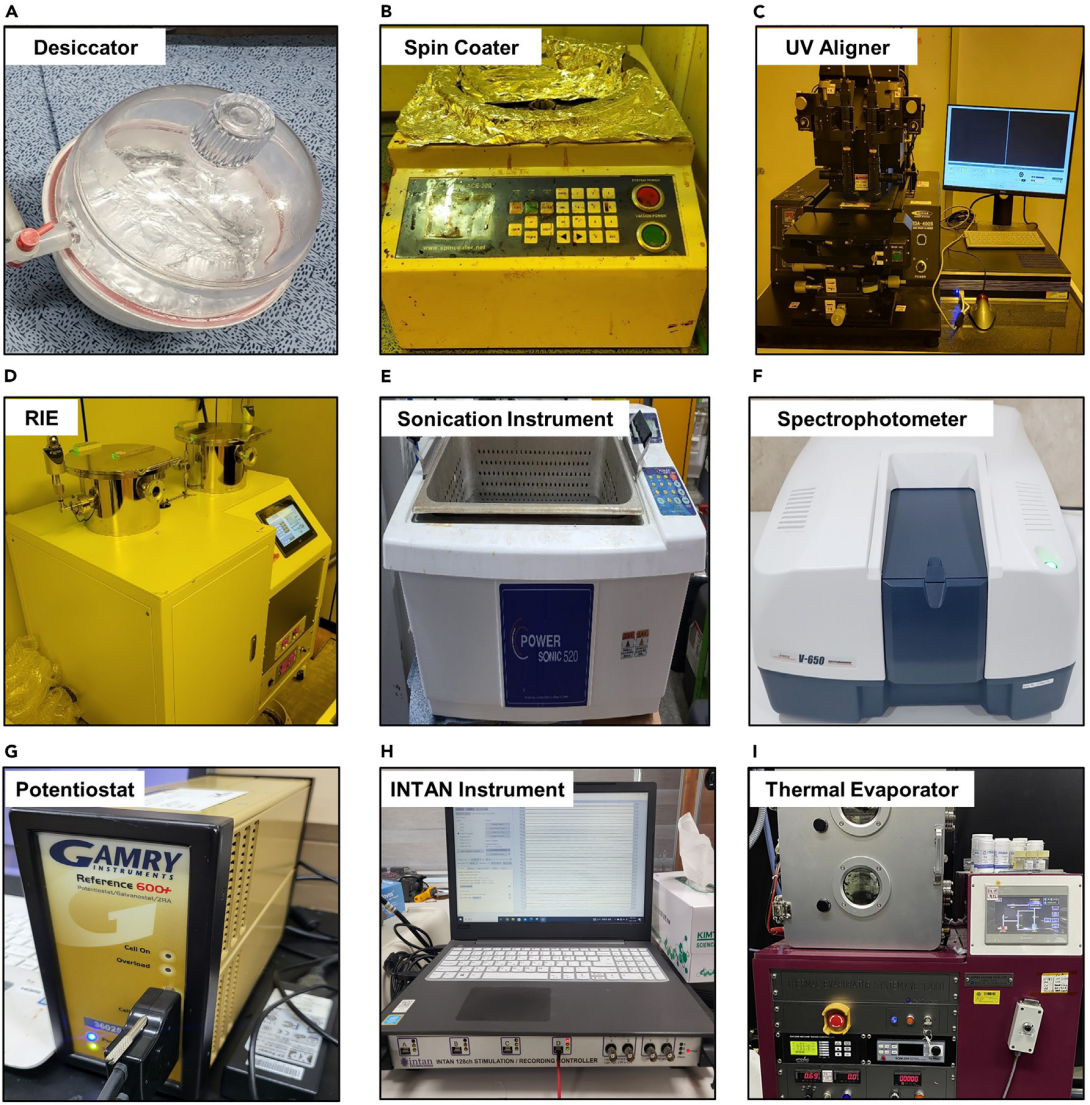
Equipment for fabrication of conventional gold (Au) electrode and transparent PEDOT:PSS MEAs with electrophysiological recording (A) Desiccator (B) Spin Coater (C) Ultraviolet (UV) Aligner (D) Reactive Ion Etcher (RIE) (E) Sonication Instrument (F) Spectrophotometer (G) Potentiostat (H) INTAN Instrument (I) Thermal Evaporator.
  d. After fixing the slide glass on the spin coater chuck while holding the vacuum, pour the prepared PDMS solution onto the glass entirely. Spin coater is shown in Figure 1B.
  e. Spin coating the poured PDMS on the glass at 2000 rpm for 50 s.
  f. For gradual curing of the PDMS, heat the coated temporary glass substrate at 70°C for 30 min and then at 110°C for 30 min on a hot plate.
  g. Cut a 25 μm thick PET film substrate to an appropriate sample size (3 cm × 2 cm) and laminate it on the completely hardened PDMS on glass.

1. Prepare a PET (Polyethylene Terephthalate) film substrate attached to the slide glass temporary substrate.
  a. Prepare a slide glass (75 mm × 25 mm × 1 mm). Then, additionally prepare a polydimethylsiloxane (PDMS) solution.
  b. After filling 32 *g* of PDMS base solution in Square Polystyrene Weighing Dishes, add 2 *g* of curing agent and mix well so that the ratio of base and curing solution is 16:1.
  c. After putting the dish containing the mixed PDMS solution into the vacuum desiccator, vacuum the chamber until the air bubbles in the PDMS disappear completely (Figure 1A).
    **CRITICAL:** If a mixed solution is used while the air bubbles in PDMS are not eliminated, the substrate yield may be reduced due to many air bubbles when curing the coated PDMS later. It is recommended to maintain the vacuum in the desiccator for at least 1 hour.

## Key resources table

**Table:** 

REAGENT or RESOURCE	SOURCE	IDENTIFIER
**Chemicals, peptides, and recombinant proteins**		

Polydimethylsiloxane (PDMS)	Dow Corning	https://www.dow.com/en-us/pdp.sylgard-184-silicone-elastomer-kit.01064291z.html#overview
Acetone	DUKSAN	CAS:67-64-4
Isopropanol (IPA)	DUKSAN	CAS:67-63-0
De-ionized (DI) water	N/A	N/A
AZ 5214E photoresist	AZ Electronic Materials	https://www.microchemicals.com/products/photoresists/az_5214_e.html
SU8-2000.5 photoresist	Kayaku Advanced Materials	N/A
AZ 300 MIF developer	AZ Electronic Materials	https://www.microchemicals.com/products/developers/mif_developers.html
SU-8 developer	Kayaku Advanced Materials	CAS:108-65-6
Poly(3,4-ethylenedioxythiophene):poly(styrenesulfonate) (PEDOT:PSS)	Clevios	81076210
Ethylene glycol (EG)	Daejung	CAS:107-21-1

**Experimental models: Organisms/strains**		

Male Sprague-Dawley rat (Albino rat – Tyrc/Tyrc, embryo 18)	Raon Bio	RjHan:SD, https://raonbio.co.kr/labanimal_rodent_Disease__SPRAGUEDAWLEY
Male transgenic mice (C57BL6 Thy1-ChR2-YFP; 8–12 weeks, 25–30 *g*)	The Jackson Laboratory	IMSR_JAX:007612
Male wild-type mice (C57BL6; 8–10 weeks, 25–30 *g*)	The Jackson Laboratory	CODE: 2498079-86

**Other**		

Slide glass	Marienfeld Superior	HSU-1000412
Desiccator	Daihan Scientific	DH.DeVS
Spin coater	Dong-AH Trade Corp.	Spin Coater ACE-200
Hot plate	Daihan Scientific	DH-WMH02503-EA
Fume hood	Sinyoung LST	LS-FNC
Razor blade	DORCO	DN52
Polyethylene terephthalate (PET) film	GFM Korea	ES301230
Photo-aligner	MIDAS SYSTEM	MDA-400S
Reactive ion etcher (RIE)	Young High Tech	N/A
Thermal evaporator	Korea Vacuum Tech	KVE-T2000
Anisotropic conductive film (ACF)	Elform	P/N HST-9805-210
Photomask	LMTEC	N/A
Ultrasonication instrument	Hwasin Tech	CODE:100-520-220
Potentiostat	Gamry Instruments	Gamry ref. 600+
Neural tissue dissociation kit	Miltenyi Biotec	130-107-677
Urethane	Sigma-Aldrich	51-79-6
Pilocarpine	Sigma-Aldrich	P6503
Optical power meter	Newport Inc., Irvine	1936-R
540 photodetector	Newport Inc., Irvine	918D
Implantable optical fiber	Thorlabs, Newton	N/A
Stereotaxic instrument	David Kopf Instruments	N/A
Intan recording system	Intan Technologies	RHD 2132
Reference electrode (Ag/AgCl)	Sanxin	6211-M
Counter electrode (platinum)	WizMAC	CE-Post-Pt-01-05-PTFE-06-80

## Step-by-step method details

### Fabrication of transparent PEDOT:PSS neural electrodes

**Timing: 4 h**

**Figure 2:**
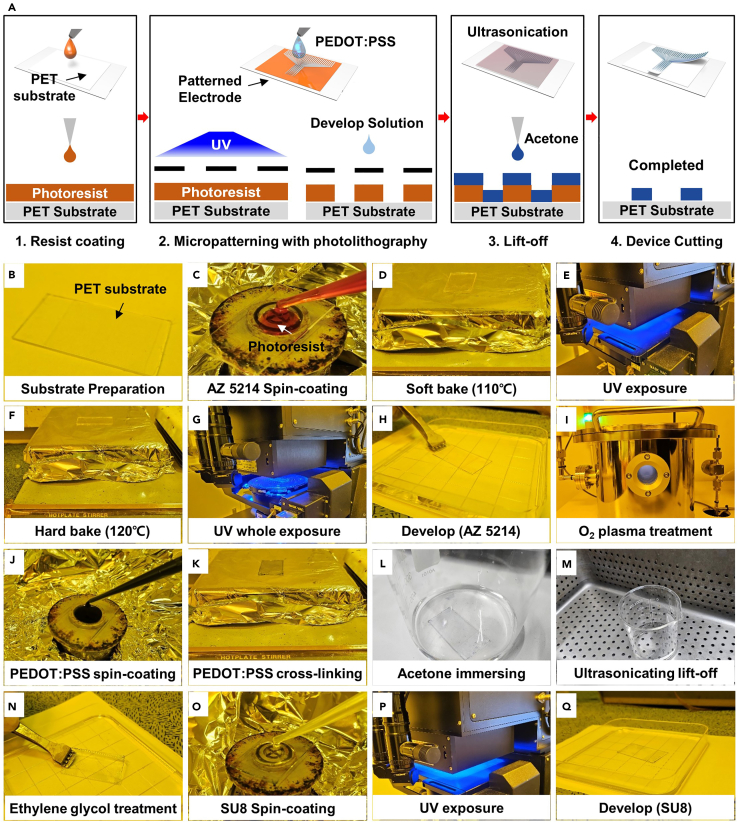
Detailed fabrication of transparent PEDOT:PSS MEAs (A) Step-by step fabrication process of transparent PEDOT:PSS MEAs. (B) Substrate Preparation (C) AZ 5214 Spin-coating (D) Soft bake (110°C) (E) UV exposure (F) Hard bake (120°C) (G) UV whole exposure (H) Develop (AZ5214) (I) O_2_ plasma treatment (J) PEDOT:PSS spin-coating (K) PEDOT:PSS cross-linking (L) Acetone immersing (M) Ultrasonicating lift-off (N) Ethylene glycol treatment (O) SU8 Spin coating (P) UV exposure (Q) Develop (SU8).

In this work, we introduce the micropatterning process of an electrode array using the conductive polymer Poly(3,4-ethylenedioxythiophene):poly(styrene sulfonate) (PEDOT:PSS) using the photolithographic lift-off method (Figure 2A). Due to the intrinsic acidity of PEDOT:PSS, researchers must be careful about direct contact with the solution when using the developer required in the photolithography process.^29^ For a higher patterning resolution, the process of removing residual photoresist material using an ultrasonication instrument during lift-off is also introduced in detail in the protocol.

1. Micropattern the electrode array on the PET substrate.
  a. Clean the laminated PET film substrate in the order of Acetone, Iso-propyl Alcohol (IPA), and deionized water (DI) (Figure 2B).
  b. Spin-coat the AZ 5214 photoresist on the PET substrate at 2000 rpm for 50 s (Figure 2C).
  c. Bake the sample with a hot plate at 110°C for 2 min (Figure 2D).
  d. After placing the sample on the photo-aligner, cover the sample with an electrode-shaped micropatterned photomask.
  e. Expose the sample for ultra-violet (UV) with UV exposure (Intensity: 14.3 W/s) for 8 s (Figures 1C and 2E).
    **CRITICAL:** If even a few small particles enter the sample on which the photomask and photoresist are applied, it can cause serious problems for microelectrode fabrication. Since the line for the external connection of the electrode is about 50 μm width, it is necessary to prevent this connection from being disconnected due to external foreign substances in advance.
  f. The photoresist coated sample partially exposed to UV is reheated on a hot plate at 120°C for 2 min and 30 s (Figure 2F).
  g. After the sample has sufficiently cooled down, place it back on the photo-aligner and expose all parts to UV (Intensity : 14 mW) for 14 s without a photomask (Figure 2G).
  h. After pouring an appropriate amount of developer (AZ 300 MIF) into the petri-dish, immerse the sample inside for 2 min and 30 s (Figure 2H).
    **CRITICAL:** If the time to develop the sample is too long, the photoresist part (the part where the resist polymer should be) that has been completely hardened by the hot plate will also disappear by the developer. The time soaked in the developer should not exceed a maximum of 4 min.
  i. After taking the sample out of the developer and washing it with DI for 20 s, remove residual water through a nitrogen blow gun.
2. Spin-coat and Lift-off the PEDOT:PSS on the PET film substrate.
  a. Put the micropatterned PET film substrate into the reactive ion etching (RIE) chamber.
  b. Perform oxygen plasma treatment on the surface (20 sccm, 100 W, 30 s) (Figures 1D and 2I).
    **CRITICAL:** If oxygen plasma treatment is not performed, PEDOT:PSS will not be spin-coated on the electrode pattern on hydrophobic surface of PET film. This process must be preceded to form an electrode array layer with a uniform thickness when coating PEDOT:PSS in a solution state.
  c. Fix the hydrophilic surface treated sample on the spin-coater chuck and apply PEDOT:PSS solution (Clevios PH 1000, ratio 1:2.5) on the surface (1500 rpm, 40 s) (Figure 2J).
  d. Crosslink the PEODT:PSS-coated sample on a hot plate at 110°C for 10 min (Figure 2K).
  e. Immerse the sample in acetone for 15 min (Figure 2L).
    **CRITICAL:** The photoresist layer that must be sacrificed by acetone is underneath the spin coated PEDOT:PSS layer. Therefore, before performing the lift-off process using ultrasonication, it is necessary to allow the acetone solution to penetrate the photoresist for at least 15 min.
  f. After putting the beaker containing the sample and acetone into the ultra-sonicating chamber, shake it moderately for 20 s while operating the chamber (Figures 1E and 2M).
  g. Take the sample out of the beaker and wash it thoroughly with IPA for 30 s.
  h. Curing the sample on a hot plate at 110°C for 10 min.
3. Perform post treatment of the PEDOT:PSS microelectrode array (MEA).
  a. Prepare the ethylene glycol (EG).
  b. After pouring an appropriate amount of EG into the petri-dish, immerse the sample for 30 min (Figure 2N).
  c. Take out the sample and wash it with IPA for 1 min.
    **CRITICAL:** The EG solution makes the treated surface slippery and has the characteristic that the residue does not easily disappear even after IPA washing. If EG residue remains, it is difficult to form a proper epoxy pattern during the post-encapsulation process to determine the recording cite, so sufficient EG cleaning time must be taken.
  d. Heat the sample on a hot plate at 110°C.
4. Encapsulate the PEDOT:PSS-EG MEA except for the recording site.
  a. After re-cleaning the fully cured PEDOT:PSS-EG MEA with IPA for 20 s, remove residual moisture with nitrogen gas.
  b. After fixing the sample on the spin-coater chuck, spin-coat the sample with UV curable epoxy (SU 8–2000.5) (3000 rpm, 40 s) (Figure 2O).
  c. Place the sample on a hot plate at 100°C for 2 min.
  d. After placing the sample on the photo-aligner, cover the photomask with the recording site pattern on the sample and expose it to UV for 4 s (Figure 2P).
  e. Place the sample on the hot plate again at 100°C for 2 min and 30 s for cross-linking.
    **CRITICAL:** The hard bake process of UV curable epoxy is a much more important task than soft bake. In the case of soft bake (2 min), exceeding the curing time does not directly affect device completion. However, if the hard bake process is not performed at all or the specified curing time is not followed, patterning of the sample is impossible.
  f. Immerse the sample in a UV curable epoxy developer for 1 min (Figure 2Q).
  g. Take out the sample and wash it with IPA for 40 s.
    **CRITICAL:** Washing UV curable epoxy that has not completely hardened with acetone or DI water may have a negative effect on the completed electrode pattern, so be sure to thoroughly wash the sample with IPA.
  h. Put the sample on the hot plate again at 120°C for 1 h to hard cure.
5. Cut the completed MEA and connect it to external neural recording equipment.
  a. While checking the interface of the completed MEA with a microscope, cut the device using a blade.
  b. Prepare anisotropic conductive film (ACF) to connect the printed circuit board (PCB) in external recording equipment with the sample.
  c. After cutting the ACF according to the number of channels of the device, place it in contact with the interconnect line of the device while bonding, and iron it at a low temperature.
  d. Carefully delaminate the finished sample from the PDMS with glass substrate based on the cut side.

### Measurement of optical, electrochemical, mechanical properties of transparent electrode array

**Timing: 8 h**

**Figure 3:**
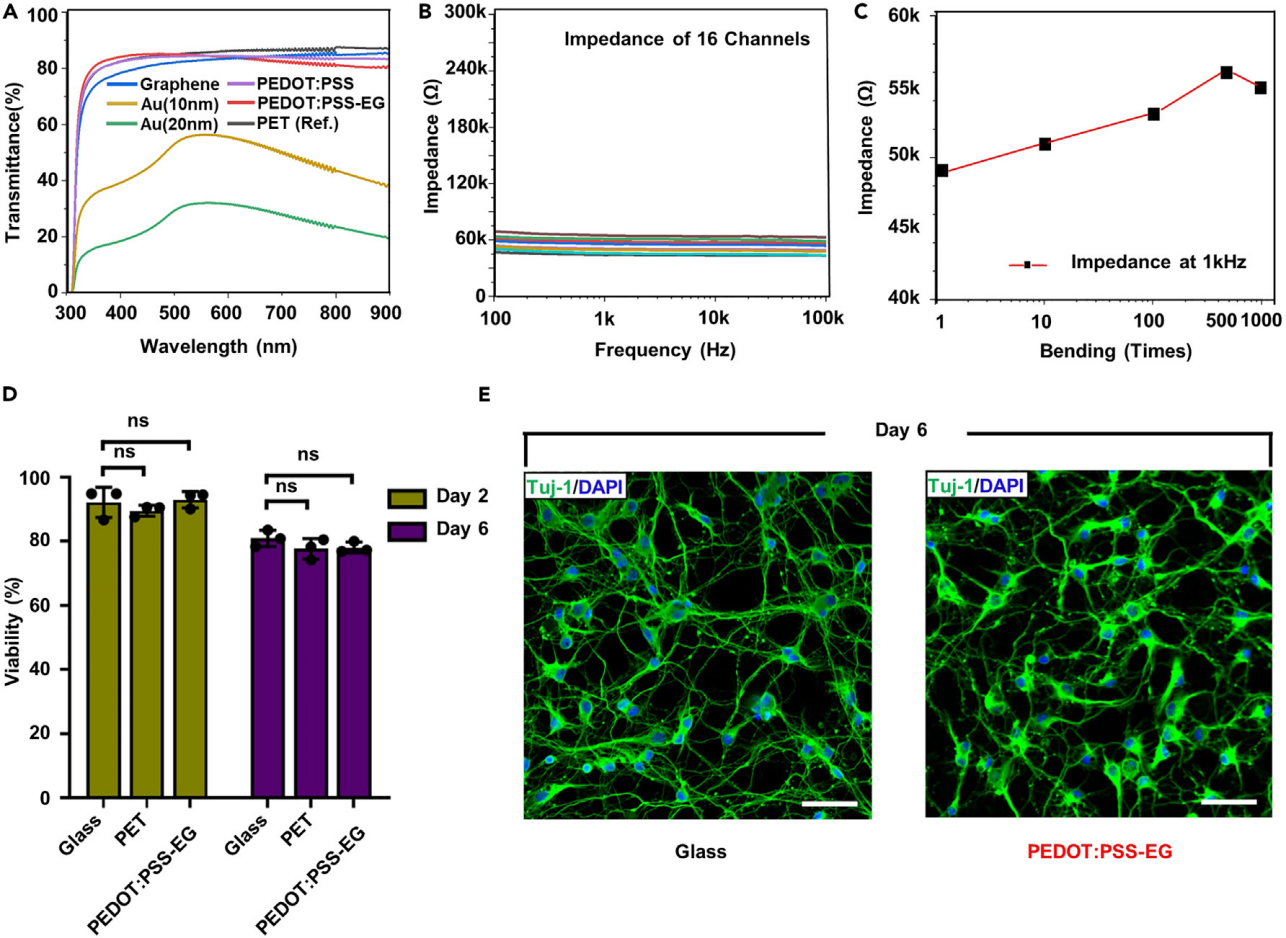
Optical, electrochemical, mechanical, and biocompatible characteristics of transparent PEDOT:PSS MEAs Figure reprinted and adapted with permission from John Wiley & Sons publications, 2021.^1^ (A) Optical transmittance plot of various candidates for transparent neural electrode array. The gray-colored line indicates the transmittance of polyethylene terephthalate (PET) substrate for reference. (B) Electrochemical impedance plot of 16 channel transparent PEDOT:PSS MEAs (Scanning Frequency : From 100 kHz to 100 Hz). (C) Electrochemical impedance variations depending on the mechanical bending test of the transparent PEDOT:PSS MEAs. (D) Viability of brain cortical neurons on various substrates (Glass, PET, PEDOT:PSS-EG) for day 2 and day 6. (E) A confocal fluorescence image of neurites (Tuj-1, Green) and cell nucleus (DAPI, Blue) on the control substrate (Glass) and PEDOT:PSS-EG at day 6. Scale bar, 50 μm.

This process is to ensure that the fabricated electrodes have sufficient neural recording feasibility before entering the *in vivo* experiment. Through this interrogation, the optical transparency, electrochemical performance, and mechanical properties of the electrode array are analyzed, and whether they are stable in the *in vivo* environment is determined. Unlike conventional Au electrode arrays, our transparent PEDOT:PSS MEAs possessed superior optical transparency, which was comparable to that of graphene (Figure 3A). In the case of electrochemical impedance, our electrode array showed 55 ± 10 kΩ in the 1 kHz frequency band in 16 channels, and it was confirmed that these electrical properties did not change even after several electrode bending (Figures 3B and 3C). Additionally, this process examined the biocompatibility of transparent PEDOT:PSS MEAs with cortical neurons cultured for 6 days. As a result, like glass without any treatment, cortical neurons cultured on the electrode also showed only negligible cell death (Figures 3D and 3E).

6. Measure the optical transparency of the PEDOT:PSS-EG film on PET substrate.
  a. Insert the PDMS-coated slide glass into the UV-visible spectrophotometer and scan the sample (for Reference Transmittance, Slew speed: 2000 nm/min) UV-visible spectrophotometer is shown in Figure 1F.
  b. Re-measure the transparent PEDOT:PSS film on PET substrate in the range of UV to visible wavelengths.
7. Measure the electrochemical impedance spectroscopy (EIS) of the PEDOT:PSS-EG MEAs on PET substrate.
  a. Prepare the phosphate buffered saline (PBS) (Sigma Aldrich, USA, 0.01 M PBS in 1 L deionized water) (pH 7.4, 36°C) in the small square dish.
  b. Prepare the reference electrode (Ag/AgCl), counter electrode (Platinum electrode), and working electrode (Transparent PEDOT:PSS-EG MEAs).
  c. Set up the impedance measurement instrument (Gamry ref. 600+ potentiostat) and attach the cable that connects the three electrodes to this system. The Gamry Reference is shown in Figure 1G.
  d. Measure the electrochemical impedance and phase of the MEAs with a frequency range from 1 Hz to 100 kHz.

**CRITICAL:** When measuring electrochemical impedance, to obtain accurate data, select a low-noise mode rather than a mode with a fast data acquisition speed.

8. Measure the electrochemical impedance of transparent PEDOT:PSS-EG MEAs according to the bending cycle.
  a. Prepare the glass rod (Diameter: 0.5 mm) and PBS solution in small square dish.
  b. Connect the PEDOT:PSS-EG MEAs to the impedance measurement instrument in the same way as the EIS measurement method.
  c. Wind the PEDOT:PSS-EG MEAs around a cylindrical glass rod (diameter: 0.5 mm) and measure the electrochemical impedance.
  d. Repeatedly record the electrochemical impedance of Transparent PEDOT:PSS-EG MEAs after bending (1, 10, 100 and 1000 times).
9. Evaluate the biocompatibility of the transparent PEDOT:PSS-EG MEAs with cultured cortex neuron.
  a. Prepare the pregnant Sprague Dawley rats (embryo 18, DBL. Co, South Korea).
  b. Sacrifice rats and create 2-dimensional neural cultures on a fabricated transparent PEDOT:PSS-EG MEAs. Extract tissue and triturate with the enzyme mixture from neural tissue dissociation kits (Miltenyi Biotec, Germary).
  c. Count isolated live neurons with a Trypan blue (GIBCO, USA).
  d. Coat the poly-D-lysine (Sigma Aldrich, USA) at a concentration of 100 μg/mL on the fabricated transparent PEDOT:PSS-EG MEAs.
  e. Satisfy the condition of culture medium, supplemented with 2% v/v of B27 Plus (Invitrogen, USA), 2 mM of Glutamax-I (Gibco, USA). Also maintain the medium temperature of 37°C with a CO_2_ incubator.
  f. Assess the cell viability at 2 and 6 days in-vitro (DIV). Treat the samples with 1X PBS (1 μM CellTrace calcein green, AM) and 15 μM propidium iodide (Sigma Aldrich, USA) at 37°C.
  g. Wash the samples with 1X PBS.
  h. Stain the cortical neurons with neuron-specific class 3 beta-tubulin (mouse anti-Tuj-1, 1:1000, T8678, Sigma Aldrich, USA) antibody.
  i. Fix the neurons on fabricated samples in 4% w/v paraformaldehyde in 1X PBS for an hour at 25°C.
  j. Wash the samples with 1X PBS for three times.
  k. Treat the samples with secondary antibody (goat anti-mouse conjugated Alexa Fluor 488, 1:1000, A-11001) in the blocking solution for 2 h.
  l. Treat the samples with 4′,6-diamidino-2-phenylindole (Invitrogen, USA, 1:1000) for 1 h after washing the 1X PBS.

Acquire the fluorescence imaging of the sample with a confocal laser scanning microscope (Carl Zeiss, LSM700).

### In-vivo seizure-like activity recording with electrodes

**Timing: 6 h**

Transparent PEDOT:PSS MEAs are implanted in a grid form in the right part of the mouse cerebral cortex for electrophysiological recording of transgenic mouse (C57BL6 Thy-ChR2-YFP; 8–10 weeks, 25–30 *g*). This electrode array is directly connected to an instrument that can record signals, and the signals received from the brain are linked to software compatible with the instrument. Then the pilocarpine was injected to the transgenic mouse to interrogate the recording feasibility of seizure activity with our electrode array. The detailed dosage of pilocarpine instruction is introduced in another related research.^30^^,^^31^ After all *in vivo* experimental processes, the signal is filtered at a frequency of 60 Hz through a notch filter, and signals in the high frequency range (300 Hz-6 kHz) are also filtered to obtain a clear signal of local field potential. Before determining whether optogenetics and electrophysiology can be integrated, this experiment was conducted to first check the recording feasibility of neural signals by measuring seizure-like activity with transparent PEDOT:PSS MEAs. Our electrode array succeeded in stably recording neural signals comparable to conventional electrode arrays, indicating that it can be applied as a tool to analyze neural activation by light stimulation.

**Figure 4:**
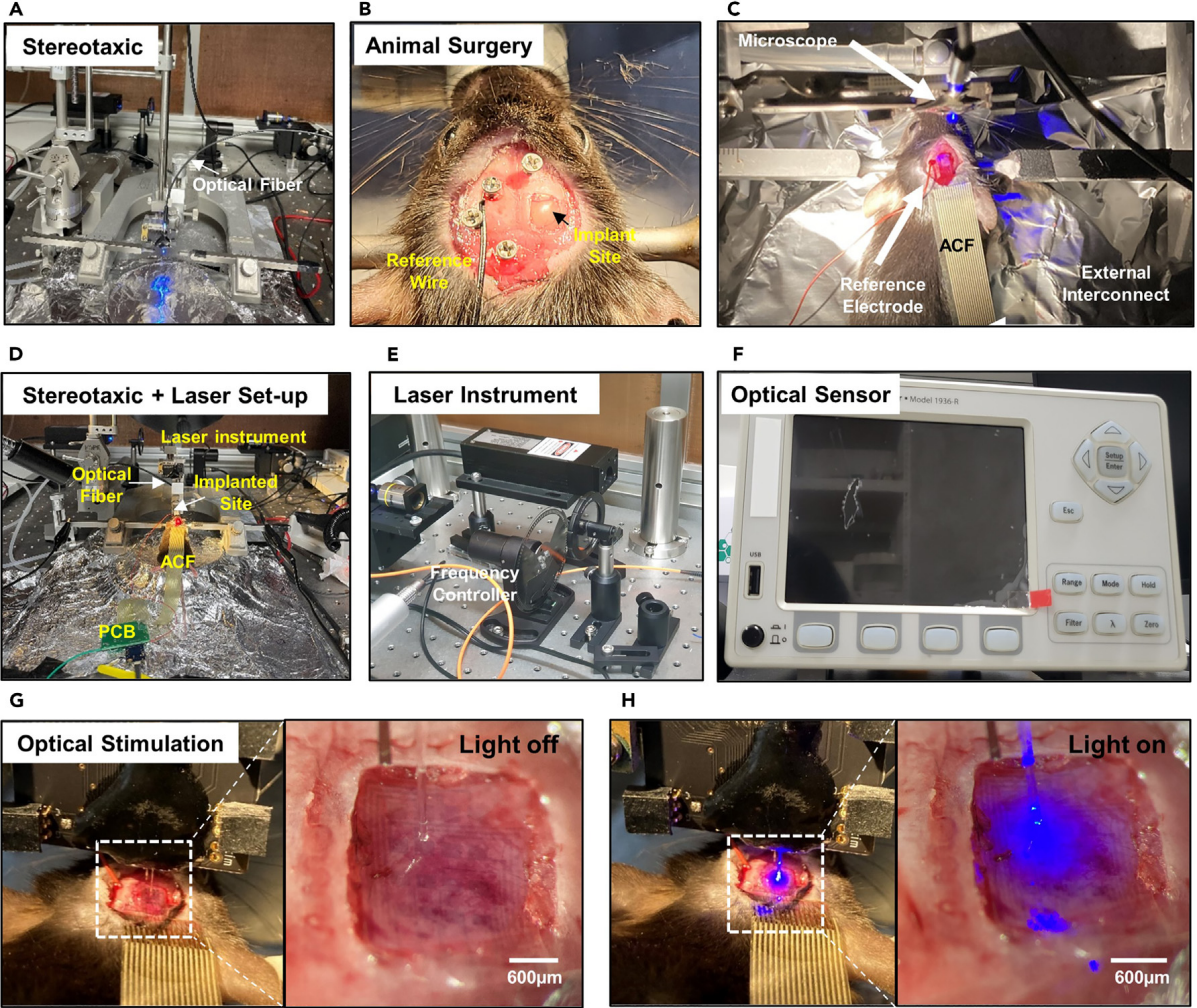
In-vivo experimental instrument set-up for electrophysiological recording & optogenetics of the brain of transgenic mouse with transparent PEDOT:PSS MEAs Figure reprinted and adapted with permission from John Wiley & Sons publications, 2021.^1^ (A) Stereotaxic instrument (B) Brain surgery of transgenic mouse (C) Stereotaxic fixation of experimental mouse and device implantation (D) Stereotaxic and laser instrument set-up for simultaneous electrophysiology and optogenetics (E) Laser instrument (F) Optical sensor for laser stimulation (G) Optical stimulation (Light off) of transgenic mouse with transparent PEDOT:PSS MEAs (H) Optical stimulation (Light on) of transgenic mouse with transparent PEDOT:PSS MEAs.
  d. Fix the anesthetized mouse on the stereotaxic, anchoring the ears and nose with the instrument.
  e. To expose the mouse’s skull, remove the fur on the head and make an incision in the scalp (Figure 4B).
  f. Using a fine electric drill, expose 2 mm horizontally and 2.5 mm vertically the brain tissue inside the skull (Right cerebral cortex of a somatosensory region) (Figure 4B).
  g. Implant the wire reference electrode (100 μm diameter), after drilling a hole in the skull to implant the wire (Left cerebral cortex of a somatosensory region) (Figure 4B).

10. Perform animal surgery and implant the transparent PEDOT:PSS-EG MEAs in the brain of animal models.
  a. Prepare the male adult transgenic mice.
  b. Anesthetize the urethane with intraperitoneal injection (1.5 *g*/kg).
  c. Prepare the stereotaxic instrument (David Kopf Instruments, USA). (Figure 4A).

**CRITICAL:** The reference electrode is effective wherever it is in the brain area adjacent to the recording electrode. To set an accurate reference potential, a metal screw is usually fixed to the skull and a metal wire is connected, but it does not matter if the reference electrode contacts with the brain tissue directly.

**Figure 5:**
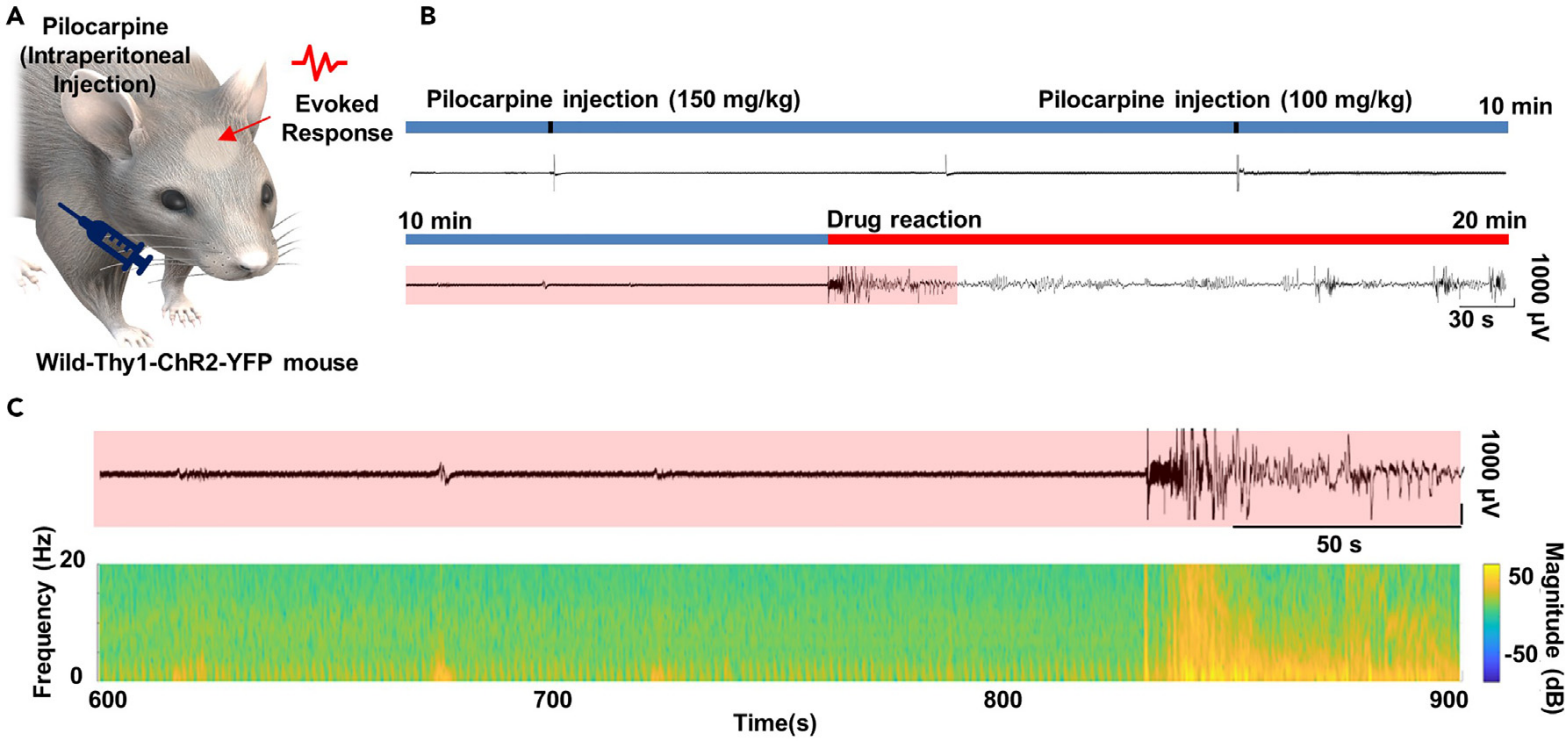
Electrophysiological recording of seizure-like activity from the pilocarpine injection with transparent PEDOT:PSS MEAs Figure reprinted and adapted with permission from John Wiley & Sons publications, 2021.^1^ (A) Schematic illustration of intraperitoneal injection of pilocarpine to the transgenic mouse. (B) Electrophysiological signal of evoked response from pilocarpine injection. (C) Evoked local field potentials (LFPs) and frequency spectra of before & after pilocarpine injection.
  d. Additionally administrate 100 mg/kg of pilocarpine with a loading time of 10 min by intraperitoneal injection (Figure 5B).
    **CRITICAL:** If pilocarpine above the appropriate level is administered to mouse, the mice may be sacrificed when acquiring neural signals due to various symptoms accompanying seizure-like activity. Therefore, the administration dose must be observed according to the weight of the experimental mouse.
  e. By analyzing raw data recorded at a sampling rate of 20 kS/s, compare the evoked potentials before and after pilocarpine administration (Figure 5C).

11. Prepare the brain recording instrument and connect it with transparent PEDOT:PSS MEAs.
  a. Prepare the brain Intan recording instrument (Intan RHD 2132 system, Los Angeles) (Figure 1H).
  b. Connect ACF with transparent PEDOT:PSS-EG MEAs.
    **CRITICAL:** If the part connecting the ACF and the electrode interconnect is not well connected, high quality neural signals cannot be obtained no matter how low the electrochemical impedance of the electrode is. If a lot of noise occurs in the electrical signal even though the electrode is not damaged, there is a high probability that there is a poor connection between the conductive film and the array interconnect.
  c. Connect the ACF end of the connected electrode array to a PCB compatible with the Intan recording instrument through heat treatment with a soldering iron (160°C) (Figure 4C).
  d. Connect the PCB to the Intan recording instrument and recording port.
  e. Connect the reference electrode to the instrument-animal model reference connector on the PCB (Figure 4C).
    ***Note:*** When the reference electrode is connected to a port from the PCB connected to the electrode to the recording instrument, the pin is automatically set.
  f. Ground the Intan instrument and individually ground the stereotaxic instrument.
  g. Lightly place the electrode on the tissue surface of the right somatosensory cortex of a mouse. The electrodes must be in direct contact with brain tissue.
  h. Use dental cement to fix all parts except the active contact area of the electrode.
12. Record seizure-like electrophysiological brain activities from the experimental mouse.
  a. After running Intan recording instrument’s software, apply a notch filter (60 Hz) and a band-pass filter of 0.1–6 kHz to the signal to be acquired.
    **CRITICAL:** In the case of local field potentials, which are recorded as the sum of all neural activities, they are meaningful for activation areas ranging from 0.1 to 10 Hz, and single neuronal spiking activity is recorded in the high frequency range above 300 Hz. When applying a band-pass filter, modify it based on the neural activity of the frequency band the researcher intends to analyze.
  b. Record the resting electrophysiological potentials of an anesthetized mouse for approximately 10 min.
  c. Administrate the 150 mg/kg of pilocarpine by intraperitoneal injection (Loading time: 5 min) (Figure 5A).

### Simultaneous optogenetics and electrophysiology

**Timing: 6 h**

**Figure 6:**
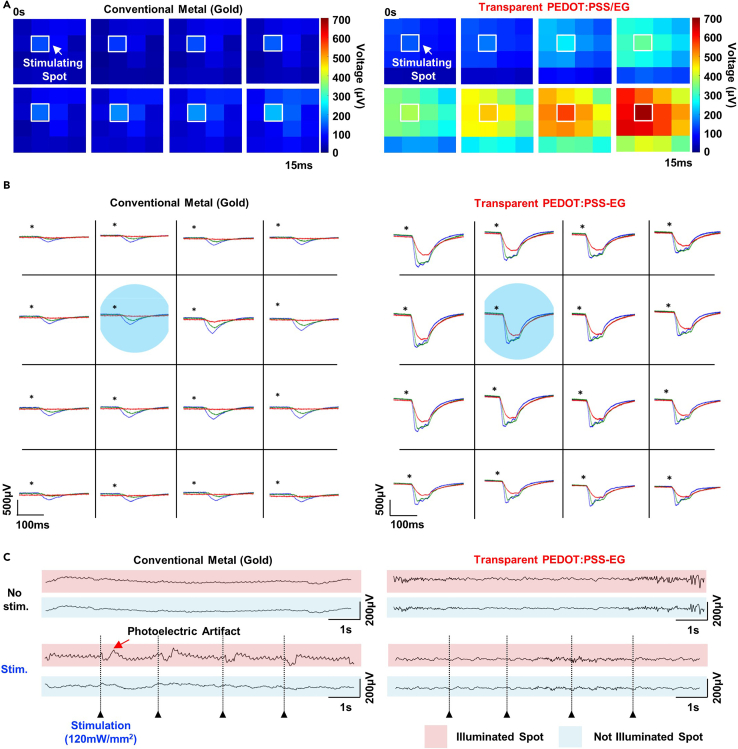
Simultaneous electrophysiology and optogenetics of transgenic mouse with conventional metal electrode array and transparent PEDOT:PSS MEAs Figure reprinted and adapted with permission from John Wiley & Sons publications, 2021.^1^ (A) Electrophysiological mapping result of 16 channel conventional metal electrode array (Left) and transparent PEDOT:PSS MEAs (Right) upon optical stimulation of transgenic mouse brain. (B) Comparison of intensity of light-evoked potentials from the light stimulation of the transgenic mouse with conventional metal electrode array (Left) and transparent PEDOT:PSS MEAs (Right). (C) Photoelectric artifact signal recording from conventional metal electrode array upon illumination of laser on the brain of wild-type mouse. Black dashed-line indicates the stimulation onset.

After confirming the recording feasibility of transparent PEDOT:PSS MEAs, we analyzed whether optogenetics and electrophysiology can be integrated. First, this protocol introduces the micropatterning process to create the same electrode shape as our electrode array using Au. Next, we showed that when our electrode array induced light directly to the electrode surface, the light-evoked potentials from the transgenic mouse were significantly higher than those of a conventional metal electrode array. This means that transparent PEDOT:PSS MEAs presented higher light transmission efficiency through electrophysiological signal mapping when light stimulation of the same intensity was applied to the somatosensory cortex of a mouse (Figure 6A). These results showed that transparent PEDOT:PSS MEAs presented superior recording efficiency even at different light intensities (237.09 (2 mW), 115.55 (1 mW), and 23.11 mW mm^−2^ (0.2 mW, square wave frequency : 0.5 Hz, duty cycle : 12.5%) (Figure 6B). Additionally, to check for light-induced artifacts in our electrodes, we applied light to wild-type mice that do not contain channelrhodopsin-2 (ChR2), a light-responsive protein, and checked whether specific artifacts were detected during neural recording. While the conventional Au electrode array showed clear photoelectric artifacts rather than local field potentials at each light stimulation onset, negligible light-based noise was measured for the transparent PEDOT:PSS MEAs (Figure 6C).

13. Fabricate a conventional Gold (Au) electrode array to compare performance with transparent PEDOT:PSS-EG MEAs.
  a. Clean the laminated PET film substrate in the order of Acetone, Iso-propyl Alcohol (IPA), and deionized water (DI).
  b. Spin coat AZ 5214-E, a positive photoresist, on the PET surface (spin speed: 2000 rpm, 40 s).
  c. Place the PET substrate coated with photoresist on the hot plate at 100°C and cure for about 2 min.
  d. Place the mask with electrode patterns on the photo-aligner and expose it to UV for about 8 s.
  e. Cure the sample with only the electrode pattern partially exposed on a hot plate for 2 min and 30 s.
  f. After placing the sample on the photo-aligner, expose all areas to UV for about 15 s without a mask pattern.
  g. After pouring an appropriate amount of AZ 300MIF developer into the petri dish, place the sample into the developer, shake the dish slightly and develop it (2 min and 40 s).
  h. Take out the sample immersed in the developer and rinse thoroughly with DI water.
  i. After removing the moisture, place the sample in the RIE chamber and treat the surface as hydrophilic using oxygen plasma for about 30 s.
  j. Place the sample in the thermal evaporator and deposit 50 nm thick Au. The thermal Evaporator is shown in Figure 1I.
    **CRITICAL:** When depositing Au, there is a possibility that the layer may be delaminated due to poor adhesion with the PET substrate. Before depositing the Au layer, it is recommended to first deposit the Cr layer to a thickness of approximately 5 nm.
  k. After preparing acetone in a glass dish, place the Au samples deposited on the patterned PET sample.
  l. Leave for 10 min until the photoresist underneath Au is dissolved by acetone, excluding the electrode pattern.
  m. Place the samples soaked for 10 min in an ultra-sonication instrument and sonicate for about 30 s.
14. Encapsulate the conventional Au electrode array with UV-curable epoxy.
  a. Wash away the Au residue blown away by sonication with IPA.
  b. Spin-coat the sample with UV curable epoxy (SU 8–2000.5) (3000 rpm, 40 s).
  c. Place the sample on a hot plate at 100°C for 2 min.
  d. After placing the sample on the photo-aligner, cover the photomask with the recording site pattern on the sample.
  e. Expose it to UV for 4 s.
  f. Place the sample on the hot plate again at 100°C for 2 min and 30 s for cross-linking.
  g. Immerse the sample in a UV curable epoxy developer for 1 min.
  h. Take out the sample and wash it with IPA for 40 s.
  i. Put the sample on the hot plate again at 120°C for 1 h to hard cure.
15. Perform animal surgery and implant the transparent PEDOT:PSS-EG MEAs in the brain of animal models.
  a. Prepare the male adult transgenic mice (C57BL6 Thy-ChR2-YFP; 8–10 weeks, 25–30 *g*).
  b. Anesthetize the urethane with intraperitoneal injection (1.5 *g*/kg).
  c. Prepare the stereotaxic instrument (David Kopf Instruments, USA) and laser instrument. The experimental set-up is shown in Figure 4D.
  d. Fix the anesthetized mouse on the stereotaxic, anchoring the ears and nose with the instrument.
  e. To expose the mouse’s skull, remove the fur on the head and make an incision in the scalp.
  f. Using a fine electric drill, expose 2 mm horizontally and 2.5 mm vertically the brain tissue inside the skull (Right cerebral cortex of a somatosensory region).
  g. Implant the wire reference electrode (100 μm diameter), after drilling a hole in the skull to implant the wire (Left cerebral cortex of a somatosensory region).

**CRITICAL:** Prior to the animal experiment, the intensity of blue laser (473 nm) to stimulate nerves must first be adjusted using an optical sensor. The light density of the laser recorded by the optical sensor is based on a fiber with a diameter of 105 μm and is the result of calibrating the intensity of light after passing through the PET substrate.

16. Prepare an optical sensor with laser equipment for optogenetics.
  a. For laser stimulation, prepare a power source instrument connected to an optical fiber with a 105 μm diameter and an optical sensor to measure the intensity of light. Photographs of laser instrument and optical sensor are presented in Figures 4E and 4F.
  b. Using an optical sensor, adjust the power source so that the light pulse density is 115.55 mW/mm and a duty cycle of 12.5%.
  c. Prepare the brain Intan recording instrument (Intan RHD 2132 system, Los Angeles).
  d. Connect ACF and transparent PEDOT:PSS-EG MEAs.
  e. Connect the ACF end of the connected electrode array to a PCB compatible with the Intan recording instrument through heat treatment with a soldering iron (160°C).
  f. Connect the PCB to the Intan recording instrument and recording port.
  g. Connect the reference electrode to the instrument-animal model reference connector on the PCB.
  h. Ground the Intan instrument and individually ground the stereotaxic to reduce motion artifacts in the animal model.
  i. Lightly place the electrode on the tissue surface of the right somatosensory cortex of a mouse. The electrodes must be in direct contact with brain tissue.
  j. Using microscopy, prepare optical fiber for laser stimulation placing directly above the electrode. Optical stimulation and magnified images of laser on/off directly on the electrode is presented in Figures 4G and 4H.
  k. When light stimulation is given at the stimulation frequency (0.5 Hz, duty cycle: 12.5%), measure the evoked potentials caused by light with an Intan recording instrument.
  l. Perform the electrophysiological recording with conventional Au MEAs light stimulation in the same way as above.

## Expected outcomes

The metal-layer free transparent electrode array using PEDOT:PSS serves as a key tool for integration with optogenetics when applied as an implantable device. Unlike PEDOT, which was deposited using conventional electroplating methods, the fabrication process introduced in this protocol can overcome disadvantages such as photoelectric artifacts and limited light transmission that may occur during optical stimulation. In this protocol, an electrochemical impedance suitable for electrophysiological recording was secured through EG post-treatment, which improved the relatively low conductivity of PEDOT:PSS. In addition, the possibility of a high-density electrode array was presented by exploiting the ultra-sonicating lift-off method to realize fine patterns within 50 μm.

In this protocol, the low electrochemical impedance, high light transmittance, and mechanical stability of the PEDOT:PSS electrode array were first analyzed. After confirming that the electrode array had sufficient performance to be inserted into a living body, seizure-like activity measurements of transgenic mice were performed to confirm the electrophysiological recording feasibility of the electrode array. As a result, we succeeded in measuring LFPs evoked by pilocarpine injection without any noise. Next, for simultaneous neural recording with optogenetic stimulation, the light transmission efficiency of transparent PEDOT:PSS MEAs and conventional Au electrode arrays was followed by comparison of neural activation in transgenic mice. It was confirmed that even at the same stimulation intensity of blue laser (473 nm), the conventional Au electrode array absorbed most of the light, so that brain nerve activity was hardly activated. In comparison, transparent PEDOT:PSS MEAs showed clear light-evoked potentials because they maintained sufficient transparency to transmit most light.

Finally, analysis of photoelectric artifacts was performed by performing recording with two electrode arrays while providing light stimulation to wild-type mice that are not affected by neural activation by light. In the case of wild-type mice, no special evoked potentials should be observed by blue laser stimulation, and the peak value seen at each onset of stimulation indicates photoelectric artifact caused by the metal layer. Notably, nerve signals contaminated by photoelectric artifacts were observed in the conventional Au electrode array, whereas only negligible noise was recorded in transparent PEDOT:PSS MEAs. These results suggest that our work can play a critical role in the integration of electrophysiology and optogenetics, making it a potential tool for future clinical applications and neural circuit analysis.

## Limitations

After fabricating the electrode array, if encapsulation to determine the recording site is not performed while the humidity in the process environment is high, performance degradation of the electrode may occur due to moisture absorption by the PEDOT:PSS layer. Although the electrochemical impedance of the electrode does not reduce performance to the extent that it has a critical effect on electrophysiological recording, it can be disadvantageous in obtaining high-quality signals, so caution is required in the device manufacturing process environment. Additionally, instability in the connection between the electrode array and the anisotropic conductive film that will lead the recording instrument can result in poor recording signal acquisition, independent of device performance.

## Troubleshooting

### Problem 1

Difficulty in patterning the microelectrode with ultrasonicating lift-off process (related to Step 1e‒1i).

If the electrode does not realize a fine pattern within 20 μm by photolithography, the most likely cause is three issues: changes in the chemical material of the photoresist, changes in the resulting ultraviolet exposure time, and developing time of the developer. If the exposure time according to the change in the properties of the solution is not properly observed, there is a possibility that all predetermined patterns may disappear or the interconnect line with the electrode may be disconnected.

### Potential solution

If the fabricated electrode pattern is observed with optical microscopy and the pattern is blurred and has a structure with a much larger width than the predetermined design, it is necessary to further increase the exposure time. Conversely, if the pattern is darker than the existing design, but the spacing between patterns appears very narrow, the exposure time should be reduced. In the case of using a developer, it does not depend on the designated developing time, and when it is visually confirmed that the red dissolved material of the photoresist has completely disappeared from the electrode surface, wash it thoroughly with DI water.

### Problem 2

The characteristics of SU 8–2000.5 change sensitively to the surrounding environment (temperature, humidity, etc.) (related to Step 4b‒4h).

During SU-8-2000.5 encapsulation to determine electrode sensing site, various unintended phenomena may be discovered. For example, there are cases where SU-8-2000.5 is not uniformly spin-coated on a completed device, or when it is not properly patterned in accordance with the developing time known as recipe.

### Potential solution

In the case of the SU-8 series negative photoresist, its properties change sensitively depending on the temperature and humidity of the surrounding environment. In other words, there are cases where proper patterning can be achieved only by using a different method from the existing recipe. When performing patterning to determine the sensing site of the electrode, if the recording site is smaller than expected, it is recommended to reduce the UV exposure time by 1–2 s. Alternatively, reducing the developing time by about 20 s is also recommended. If SU-8 is not uniformly spin-coated on the electrode, it is recommended to first change the electrode surface to a hydrophilic surface through mild O_2_ plasma treatment.

### Problem 3

Electrical noise from the external equipment from the experimental setup (related to Step 11f).

Despite sufficient grounding of the Intan recording instrument and proper insertion of the reference electrode, unwanted electrical noise can frequently occur during neural recording. This is most likely due to the possibility of noise penetration by external electronic equipment or a defect in the PCB compatible with the Intan recording instrument.

### Potential solution

If electrical noise continues to occur even after sufficient grounding, it is highly likely that the PCB itself has a poor connection. Under the premise that electrode performance is perfectly guaranteed, all Intan recording instruments should be reexamined for poor connections and external electronic devices should be placed far away.

### Problem 4

When stereotaxic coordinates are not specified accurately (related to Step 11g).

When implanting electrodes into the brain of a transgenic mouse, be sure to check the stereotaxic coordinates of the brain region to be inserted before performing the experiment. If some time has passed since the electrodes were placed on the surface of the brain, the moisture in the brain surface tissue may dry and stick to the electrodes. In this case, be aware that tissue damage or scar may occur if the electrode is immediately removed from the brain surface.

### Potential solution

If researchers want to change the stereotaxic coordinates after the electrocorticography grid is raised to the surface of the brain, first sprinkle enough PBS droplets around the electrode to supply moisture to the surrounding area. Afterwards, considering the narrowing between the electrode and the tissue, the electrode should be slowly removed from the surface placed again on the desired region.

### Problem 5

Distinguishing between external noise and pilocarpine evoked potentials (related to Step 12c).

When performing pilocarpine injection to check electrophysiological recording feasibility, data may be obtained that makes it impossible to clearly distinguish whether the measured signal is an evoked potential caused by pilocarpine or simple noise.

### Potential solution

The clearest way to distinguish between external noise and a neural signal coming from the brain is to analyze the analog signal in the frequency range. It is possible to distinguish between noise and absence seizures using magnitude analysis by frequency at the point when brain activity caused by pilocarpine begins to appear.

## Resource availability

### Lead contact

Further information and requests for resources and reagents should be directed to and will be fulfilled by the lead contact, Ki Jun Yu (kijunyu@yonsei.ac.kr).

### Technical contact

For technical questions, please contact, Young Uk Cho (phdyoungukcho@gmail.com).

### Materials availability

This protocol does not include any introduction to the development of new materials or new chemicals.

### Data and code availability

All data reported in this paper will be shared by the lead contact upon request.

No code was generated in this study.
